# Self-Association of Antimicrobial Peptides: A Molecular Dynamics Simulation Study on Bombinin

**DOI:** 10.3390/ijms20215450

**Published:** 2019-11-01

**Authors:** Peicho Petkov, Elena Lilkova, Nevena Ilieva, Leandar Litov

**Affiliations:** 1 Faculty of Physics, Atomic Physics Department, Sofia University “St. Kliment Ohridski”, 5 J. Bouchier Blvd, 1164 Sofia, Bulgaria; 2 Institute of Information and Communication Technologies at the Bulgarian Academy of Sciences, Acad. G. Bonchev Str., Block 25A, 1113 Sofia, Bulgaria; 3 Institute of Informatics and Mathematics at the Bulgarian Academy of Sciences, Acad. G. Bonchev Str., Block 8, 1113 Sofia, Bulgaria

**Keywords:** antimicrobial peptides, self-association, aggregation, promotion of folding

## Abstract

Antimicrobial peptides (AMPs) are a diverse group of membrane-active peptides which play a crucial role as mediators of the primary host defense against microbial invasion. Many AMPs are found to be fully or partially disordered in solution and to acquire secondary structure upon interaction with a lipid membrane. Here, we report molecular dynamics simulations studies on the solution behaviour of a specific AMP, bombinin H2. We show that in monomeric form in water solution the peptide is somewhat disordered and preferably adopts a helix-loop-helix conformation. However, when more than a single monomer is placed in the solution, the peptides self-associate in aggregates. Within the aggregate, the peptides provide each other with an amphipathic environment that mimics the water–membrane interface, which allows them to adopt a single-helix structure. We hypothesise that this is the mechanism by which bombinin H2 and, possibly, other small linear AMPs reach the target membrane in a functional folded state and are able to effectively exert their antimicrobial action on it.

## 1. Introduction

Antimicrobial peptides (AMPs) are a crucial part of the nonspecific innate immunity of all eukaryotes to microbial invasion [1,2,3]. They are a diverse group of compounds, displaying various types of structures, including α-helices, β-sheets or cyclic structures. Nonetheless, AMPs share some general characteristics: they consist of 6–100 amino acids, usually are cationic and have an amphiphilic nature. Most importantly, AMPs are generally membrane active peptides that interact with target membranes and can cause cell death through various mechanisms [1,4,5]: they can disturb the membrane by inducing thinning, altering its curvature or fluidity, modifying the transmembrane electrochemical gradient, and inducing pore formation [5,6].

Experimental data and theoretical studies have shown that many AMPs are partially or fully disordered in solution and acquire their functional secondary structure upon interaction with the amphiphilic membrane–solvent interface [6,7,8,9,10]. The issue of whether secondary structure content is necessary for successful attack on the bacterial membrane is still controversial [11,12,13,14]. In addition, the activity of many AMPs depends on the peptide concentration and it has been shown that AMPs self-organise and cooperatively form pores upon interaction with a lipid bilayer [11,15,16,17,18,19,20,21,22,23]. AMP aggregation in solution and in the absence of a membrane is not well studied, while this certainly is the first stage of their activity pathway whose significance is that way largely underestimated. This is all the more important in view of recently reported observations [24] about the decrease in AMPs activity upon aggregation due to increased membrane-embedding free-energy costs. In the same time, in a very recent paper [25], the authors demonstrated that not only was α-helical structure necessary for the antimicrobial action of a specific AMP—halictine-1—but also the *“mechanism of the peptide mode of action probably involves formation of peptide assemblies (possibly membrane pores), which disrupt bacterial membrane and, consequently, allow membrane penetration”*.

We consider the issue of whether aggregation is beneficial or unfavourable for AMPs efficient action is still controversial and probably depends on the type of AMP. The design of AMPs with predefined properties requires a detailed and precise understanding of their mechanism of action that allows identification of crucial for each step of this mechanism residues to be targeted for optimisation. Therefore, the process of peptide aggregation has to be especially taken into account in designing potent AMPs for therapeutic purposes, with the attention focused on aggregation-prone amino acids and amino-acid motifs.

In recent papers [26,27], we discussed the process of peptide aggregation and its effect on the monomer’s secondary structure in the case of one intrinsically disordered AMP (indolicidin) and another linear α-helical AMP, magainin 2. Here, we report molecular dynamics (MD) simulations studies on the solution behaviour of a specific AMP, bombinin H2 (amino acid sequence IIGPVLGLVGSALGGLLKKI), secreted by the skin of the European *Bombina variegata* frog species. It is active against both Gram-positive and Gram-negative bacteria, and also fungi. In addition, bombinin H2 peptides display hemolytic activity at relatively low concentrations [28]. These peptides are rich in glycine (25%), which allows them to adopt different conformations [29]: α-helical, partially disordered and even β-sheet structures [28]. However, at physiological salt concentrations and pH levels bombinin H2 peptides usually form classical amphiphilic α-helices at the lipid bilayer [29].

In this work, we demonstrate that the monomeric bombinin H2 peptide in water solution is somewhat disordered and preferably adopts a helix-loop-helix conformation. When multiple peptide chains are present in the solution, they rapidly self-associate in aggregates. Aggregation promotes further folding of bombinin H2 by mimicking the water–membrane amphipathic interface. Individual monomers adopt a single-helix structure and are stabilised in this conformational state.

## 2. Results

### 2.1. Dynamics of the Bombinin H2 Monomer in Solution

We first focus on the dynamics of an isolated bombinin H2 monomer in water solution. We started the production MD simulation from a helix-loop-helix conformation of the peptide. The initial experimental all α-helical structure rapidly adopts this state within the 10 ns NPT equilibration simulation. The bend is at Gly${}^{10}$-Ser${}^{11}$.

The peptide explores various conformations multiple times during the first 1.2 μs of the simulation. During the last 800 ns of the simulation, bombinin H2 stabilises in the collapsed helix-loop-helix conformation.

**Figure 1:**
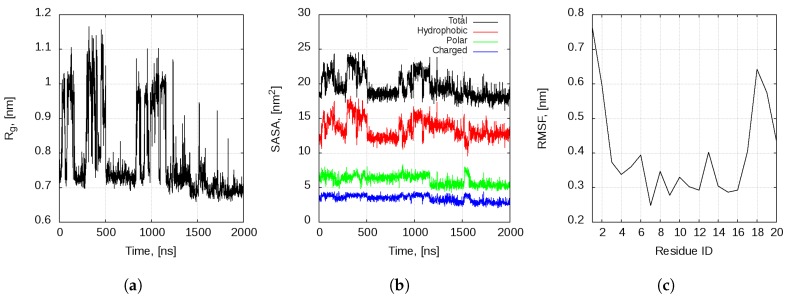
Time evolution of the peptide: (**a**) gyration radius; (**b**) SASA; and (**c**) root mean square fluctuations per residue of monomeric bombinin H2 in water.

The cluster analysis reveals six clusters, accommodating about 90% of the conformations, and a number of smaller clusters without statistical significance, each of them containing less than 2% of the structure (Figure S1). In Figure S2, the cluster-size distribution and the centroids of the four largest clusters are shown. The first one, with 1132 conformation states (almost 57% of all states), corresponds to the initial structure—a rather regular V-shaped helix-loop-helix conformation. The fully folded (single-helix) conformations form the second-largest cluster, encompassing some 15% of the structures. Clusters 3 and 4 again correspond to V-shaped helix-loop-helix conformations with a tendency towards unfolding of the N-terminal helical part. Clusters 5 and 6 (not shown) follow the same tendency and the remaining clusters, summing up to about 9% of all conformations, have no statistical significance as each of them does not exceed 2% of the states. There are numerous transitions between the two main conformational states—a single classical linear α-helix and helix-loop-helix. This can be clearly seen from the evolution of the gyration radius of the peptide (Figure 1a).

The peptide behaviour seems to be driven by the hydrophobic effect. Examination of the solvent accessible surface area (SASA) of the peptide (Figure 1b) reveals that, while the charged and polar amino acid sidechains are solvent-exposed in both states, the compact helix-loop-helix conformation reduces the solvent exposure of the hydrophobic residues in the middle of the peptide (Val${}^{5}$, Leu${}^{6}$, Val${}^{9}$, Leu${}^{13}$ and Leu${}^{17}$).

The most flexible part of the peptide molecule are the two termini, and especially the N-terminus (residues Ile${}^{1}$–Val${}^{5}$). This is reflected in the plot of the root mean square fluctuations (RMSF) per amino acid residue (Figure 1c). The higher flexibility of these amino acid residues is consistent with the experimental data by Zangger et al. [29]. They observed that, even in a lipid bylayer, bombinin H2 has a well defined α-helical structure only between residues Val${}^{5}$ and Lys${}^{17}$. Henceforth, this is the amino acid range that we use to determine if the peptide is in the single-helix or helix-loop-helix state.

**Figure 2:**
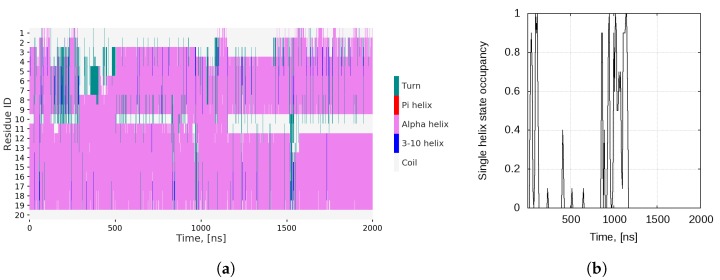
(**a**) Secondary structure; and (**b**) occupancy of the single *α*-helix conformational state, averaged over 10 ns windows, of monomeric bombinin H2 in water.

The secondary structure plot (Figure 2a) also demonstrates the multiple transitions between the two main conformational states. Not only does the single helix break at Gly${}^{10}$–Ser${}^{11}$, but the peptide does explore some very disordered conformations, where the whole N-terminal or C-terminal part of the molecule is not folded (e.g., the intervals 120–500 ns, 960–975 ns and 1520–1532 ns). Population of such disordered states is in agreement with experimental data [29].

The single-helix state is fairly regularly visited, but apparently it is not very stable, since the peptide does not remain in it for long intervals. Figure 2b shows the occupancy of this state, averaged over 10 ns windows. After the 1157th ns, this conformation is no longer adopted at all and the structure transitions permanently to the helix-loop-helix conformational basin. On average, the peptide resides in a classical linear helix conformation in 11.7% of the trajectory frames.

**Figure 3:**
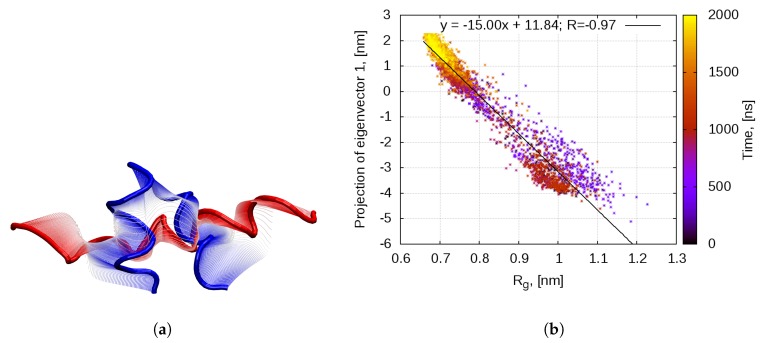
(**a**) Bombinin H2 peptide backbone motion along the first principal component; and (**b**) correlation between the projection of first principal component and the gyration radius of the peptide.

Cartesian principal component analysis (PCA) on the backbone of the peptide confirms that the main mode of motion is indeed the bending of the linear helix (Figure 3a). The projection of the eigenvector with the largest eigenvalue (Principal Component 1 (PC1)) correlates perfectly with the gyration radius of the molecule (Figure 3b). In the first half of the simulation, the peptide visits conformations in both basins—the linear single-helix (R${}_{g}\in \left[0.9,1.1\right]$ nm, PC${}_{1}\in \left[-4,-2\right]$ nm) and the compact helix-loop-helix (R${}_{g}\in \left[0.55,0.80\right]$ nm, PC${}_{1}\in \left[-0.5,2.5\right]$ nm). In the second half of the simulation, the peptide permanently transitions to the second basin. This demonstrates that PC1 corresponds entirely to the compactification of the peptide, in order to reduce the solvent exposure of hydrophobic residues.

### 2.2. Peptide Self-Assembly in Water Solution

Our working hypothesis is that small linear AMPs self-associate into larger compounds (aggregates) prior to attacking the bacterial membrane. Therefore, we studied the evolution of a set of 27 bombinin H2 monomers in water, the peptides being placed in a cubic simulation box sized 15 × 15 × 15 nm${}^{3}$ (see Section 4). The box size meets the requirement for a minimal distance of 5 nm between two neighbouring monomers, leading to a simulated system of over 325,000 atoms, with a bombinin H2 concentration of 13 mM. The simulated evolution was 2.2 μs. In a somewhat similar study [30], multiple compositions of tetrapeptides were simulated in a cubic box for 100 ns per composition but at about two times higher concentration.

We observed two processes that take place simultaneously when more than one single bombinin H2 peptide chain is placed in the simulation box: a gradual self-association process of the peptides into larger aggregates with an increasing number of monomers and a decreasing number of aggregates, respectively, until all 27 monomers formed a single aggregate that remained stable until the end of the simulation, and a self-organisation within this aggregate that promotes folding of the individual peptide chains.

#### 2.2.1. Aggregation

**Figure 4:**
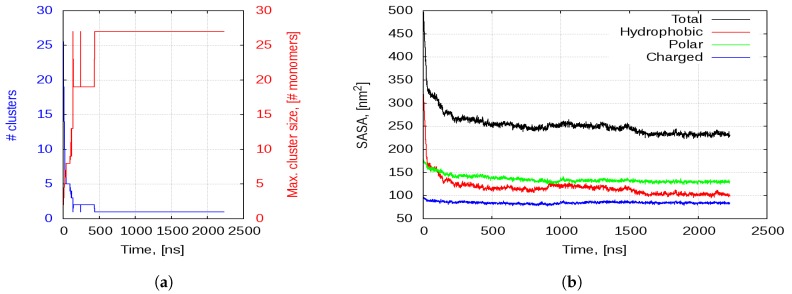
(**a**) Number of separate clusters/aggregates in the solution (blue curve) and maximal size of the clusters (red curve); and (**b**) SASA of all peptides in the solution.

Although each of the peptide chains has a +2e net positive charge, the peptides do not repulse each other, but rather start to very rapidly aggregate. Figure 4a shows the number of aggregates that are formed in the simulation box and the number of peptide chains participating in the largest aggregate. The number of aggregates starts at 27, as we have 27 peptide chains separated in space. Then, it decreases very quickly as smaller aggregates lump together and the maximal aggregate size grows.

**Figure 5:**
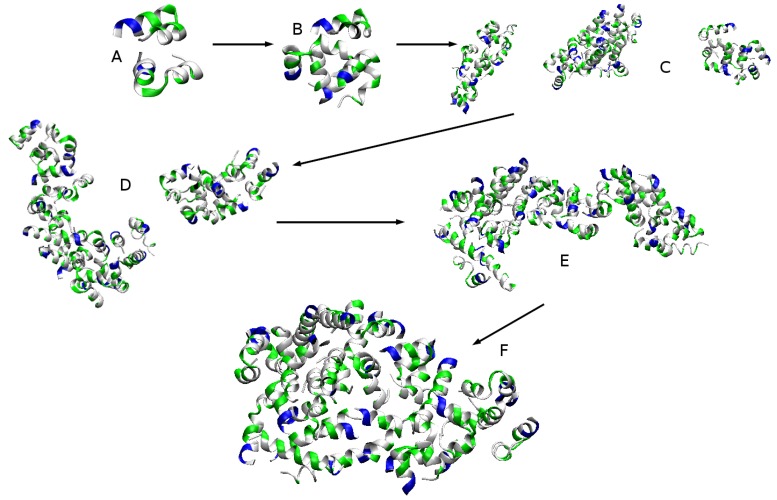
Time evolution of the self-assembly process of bombinin H2 peptides. (Charged residues are coloured in blue, polar in green, and hydrophobic in silver.)

Within the first 4–5 ns of the trajectory, the first few dimers and trimers are formed (Figure 5A). At 15–16 ns, almost all of the peptides are part of a dimer or a trimer and the first tetramer appears (Figure 5B). This is followed by the formation of pentamers at 18 ns. By the 28 ns, there are no monomers and two hexamers assemble. Within the next 50–60 ns, the smaller oligomers aggregate further into medium sized aggregates and stabilise. At about 120 ns, there are only three aggregates—a hexamer, an octamer and a 13-mer—and 20 ns later the hexamer joins the 13-mer to form a 19-mer (Figure 5C,D, respectively). By the first 0.5 μs, all of the peptides are forming one very large aggregate, which remains stable to the end of the simulation but changes in shape and becomes more compact and globular (Figure 5E,F).

The self-association process is entirely driven by the hydrophobic effect. As evident in Figure 4b, the total SASA of the peptides drops sharply with the formation of the first aggregates and continues to decrease as they consolidate into larger and larger structures. This behaviour is almost fully accounted for by the decrease in hydrophobic SASA. As shown in Figure 5A,B, the individual chains associate in such a way as to orient their hydrophobic surfaces to face each other. This reduces their exposure to the solvent. As smaller aggregates join together into larger ones, the total SASA gradually drops to about 250–260 nm${}^{2}$ and remains at that level for the next microsecond. Then, after 1.5 μs, it descents further to about 230 nm${}^{2}$ as the aggregate compactifies into a more globular shape. This is also associated with the same decrease of about 20 nm${}^{2}$ in the hydrophobic SASA. The SASA of the polar amino acid residues does not change drastically, except for the first few ns. After that, there is a slight decrease in this property as individual peptide chains undergo conformational changes within the structure of the aggregates. After the first microsecond, the polar SASA remains unchanged. Moreover, the SASA of the charged lysines stays practically constant through the simulation at a level 27 times the charged SASA of a monomer in water. This means that virtually all lysines are at the aggregate surface and are completely solvent exposed.

Different amino acid residues exhibit different propensity towards aggregation. As demonstrated in the present study and also observed by Kuroda et al. [30], the aggregation is driven by the hydrophobic effect—the interplay of the Van der Waals interactions between the amino acids plus the entropic contribution to the solvent on the one hand and the Coulomb repulsion on the other hand. The balance between these different forces determines the maximal size of the aggregate. However, exploring this issue is beyond the scope of the present work. In [30], it was reported that Ile, Leu, Val and Met, along with the aromatic Phe, Tyr and Trp, tend to aggregate very quickly into large amorphous clusters. Bombinin H2 does not contain any aromatic residues, but has five leucines, three isoleucines and two valines, i.e., half of all amino acid residues are highly aggregation-prone. Apparently, until reaching certain (saturation) number of monomers in the aggregate, the attractive Van der Waals interactions among these ten hydrophobic residues tend to take over the electrostatic repulsion between the net positive charges provided by the two lysines in each monomer.

#### 2.2.2. Aggregation-Driven Folding

**Figure 6:**
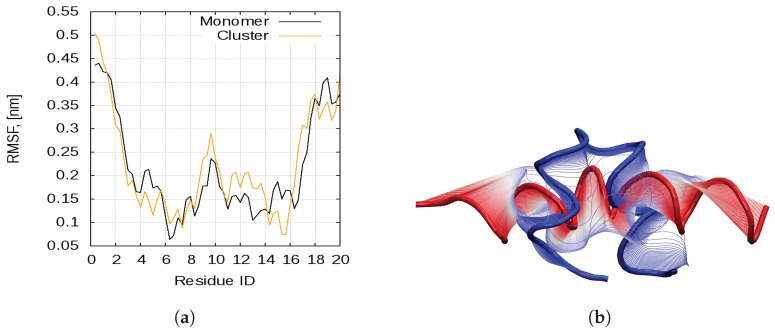
(**a**) Backbone RMSF along the first eigenvector of the PCA analysis of the bombinin H2 peptides in monomeric form (blue curve) and in a concentration/aggregate (red curve); and (**b**) peptide backbone motion along the first principal component for the simulation in concentration.

Almost from its very beginning the aggregation is accompanied by another remarkable process—some peptide chains transition from the compact helix-loop-helix to the linear single-helix state. Indeed, when cartesian backbone PCA is performed on the trajectory of all 27 peptides, the principal mode of motion corresponds to straightening and bending of the linear helix at Gly${}^{10}$-Ser${}^{11}$ (Figure 6b). This mode matches PC1 of the monomeric bombinin H2 dynamics (Figure 6a). The RMS fluctuations per C${}_{\alpha }$ atom, generated by the first eigenvector for the two trajectories, are shown on Figure 6a. As evident, the two first PCs involve motion of the same atoms.

The first transitions to a linear helix state happen shortly after the aggregation onset—chains *V* and *a* that form a dimer spontaneously adopt the single-helix state within the first 25 ns of the simulation (Table 1). Shortly thereafter, a third helix straightens (chain *W*). For the next more than 330 ns, no further transitions are observed, until chain *F* snaps from the helix-loop-helix state to the single-helix state. Another 330 ns later, a fifth peptide (chain *E*) adopts this conformation, followed by the folding of three more chains (chains *A, B* and *D*) at about 900 ns. By 1.5 μs, the last chain (chain *J*) transitions to the single-helix state.

It should be noted that, in the aggregates, once a peptide transitions from a helix-loop-helix to the linear single-helix conformation, it remains stable in this state. This can be observed in Figure S4, which shows the occupancy of the single-helix state for each of the 27 peptide chains. Therein, one sees that, during the 2 μs simulation, all monomers but two (chain *U*, and—with small exceptions—chain *Z*) explore linear conformations towards the single-helix state. Comparing chains *A, B, D, E, F, J, V, W,* and *a* with the behaviour of the single monomer in solution in Figure 2b, it becomes obvious that the straightening of the helices in the aggregates has irreversible character. Even when the single-helix state occupancy drops sharply (i.e., in chains *E* or *J* right after 1.5 μs), the peptide chains remain in a linear state. The helix occupancy decreases, because the helix bends somewhat at Leu${}^{6}$-Gly${}^{7}$, Val${}^{9}$-Gly${}^{10}$ or Leu${}^{13}$-Gly${}^{14}$ or the helix turns widen a little bit. However, as seen in Figure S5, the gyration radius remains stably in the linear conformation domain, $R_{g}\in \left[0.9:1.05\right]$ nm.

The aggregation is associated with the formation of intermolecular contacts between the amino acid residues in different peptide monomers. In Figure S3, the number of intermolecular contacts per frame for each of the amino acid residues in the bombinin H2 molecule is shown for the single-helix and the helix-loop-helix states. In general, the single-helix structured monomers tend to build more contacts (about 19% more) with the neighbouring peptides—this is the case for 13 out of 20 residues in the single-helix peptides. For two residues—Leu${}^{17}$ and Lys${}^{19}$—the number of contacts is the same, and only three residues build more contacts with the neighbouring structures while in a helix-loop-helix state. Note that the tendency towards an increase of the contacts number in a single-helix state is particularly pronounced in Leu/Ile and Gly residues: in five out of eight Leu/Ile residues, the single-helix state contacts are 30–300% more than those in the helix-loop-helix state, and in four out of five Gly residues, this increase is even stronger. These are exactly the residues associated with AMPs’ antimicrobial activity. Cationic Gly-Leu-rich peptides are hemolytic and very potent against microorganisms [31]. In the design of peptide analogues with higher antimicrobial activity, the increase of the net positive charge and of the hydrophobicity are often targeted through Lys and Leu substitutions (see, e.g., [32]). This, together with the observed correlations between the aggregation propensity and the antimicrobial activity gives one more reason for a detailed research on AMPs’ aggregation as an important and possibly decisive part of their antimicrobial action.

## 3. Discussion

The vast majority of AMP research focuses on the interaction of the peptides with target membranes and here molecular simulations play a particularly important role. However, studying the behaviour of AMPs in water solution, prior to their interaction with the membrane, is in our assessment an undervalued problem when trying to understand the AMP mechanism of action. It was shown recently that MD simulations of the interaction of AMPs and lipid bilayers are very sensitive to the initial simulation setup, including the initial conformation of the AMP and its placement relative to the membrane [11]. In their work, Wang et al. studied the interaction of a synthetic AMP, CM15, with a neutral POPC and a negatively charged POPG:POPC membranes. They performed multiple MD simulations starting from different initial conformations of the CM15 peptide—either a random-coil or a pre-folded α-helical conformation. Somewhat unexpectedly, they found that, when the AMP was pre-folded, its binding and insertion in both membranes was reduced, compared to when the simulation starts from a random-coil conformation of the peptide. Their results demonstrate the significance of the initial conformation of the AMP when simulating its interaction with a target membrane.

**Figure 7:**
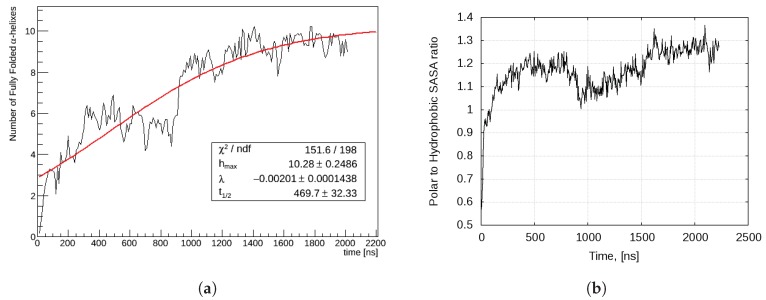
(**a**) Number of monomers in the single-helix state; and (**b**) polar to hydrophobic SASA ratio.

The experimental evidence suggests that the functional state of bombinin H2 and, in general, of the predominant part small linear AMPs is that of a single α-helix. Our investigations show that this conformation is supported in a solution only within a self-assembled aggregate, with a gradual increase of the monomers that adopt it, as depicted in Figure 7a. We observe that, at the beginning, the process is very fast—first monomers adopt a single-helix conformation within the first 100 ns, while in small aggregates (dimers or a tetramer). Next, but much later, about 400 ns, a peptide within a 19-mer straightens. The next four transitions happen around 700 and 900 ns, all within the already formed 27-mer. The process saturates at about 1.5 μs, on the level of 1/3 of the monomers (9 out of 27), and is reasonably well approximated by a sigmoid-type curve

(1) $$N_{h}\left(t\right)=\frac{h_{max}}{1+e^{\lambda (t-t_{1/2})}},$$

where $N_{h}\left(t\right)$ is the number of straightened monomers as a function of time; $h_{max}$ is the asymptotic value corresponding to the maximal number of such monomers; $t_{1/2}$ is the half-saturation time, i.e. the time when half of the asymptotic value is reached; and λ is a shape parameter. The values of these parameters for the investigated dynamics are given in the data-box in Figure 7a.

Thus, the bigger is the aggregate, the slower does the conformational transition occur; however, several transitions might then follow within a short interval. Apparently, the conformational transition from a more compact to a less compact state of a monomer within the aggregate has not only local consequences but is also associated with large-scale rearrangements of individual peptide chains within the aggregate. One might speculate that such a behaviour is rooted in the associated free energy changes. A direct verification of this hypothesis would be very involved if at all possible. However, some insight might be gained by examining the different SASAs behaviour and, in particular, the ratio between polar and hydrophobic ones, $\sigma_{p/hphb}\left(t\right)=A_{polar}\left(t\right)/A_{hydrophobic}\left(t\right)$ (Figure 7b). Note that, while these two SASA curves appear rather smooth and (almost) monotonic, their ratio proves very sensitive to even small but coincidental fluctuations in the respective values.

There is a clear correlation between the evolution of the number of single-helical monomers and $\sigma_{p/hphb}\left(t\right)$ (Figure 7a,b). Each act of monomer straightening is actually preceded by a noticeable decrease (drop) in the hydrophobic SASA and a soft decrease in the polar one and is then followed by a continuing polar SASA decrease, together with a local increase in the hydrophobic one. This is manifested in the nonmonotonic character of $\sigma_{p,hphb}\left(t\right)$ (Figure 7a). When this transition happens within a small aggregate (events in the first 100 nanoseconds and around 400 ns), the original positive slope is rapidly re-gained, while by the transition around the 700 ns within the final 27-mer this happens only partially, to be succeeded by a very pronounced drop, associated with the almost simultaneous conformational transition of three more monomers, within the already rather compact aggregate. After that, $\sigma_{p,hphb}\left(t\right)$ becomes an increasing function again and we see no indications for further transitions or major rearrangements.

This all can be understood as an interplay between the self-assembly and folding-promotion processes, which contribute differently to the formation of the various SASA figures. As a result, due to large-scale rearrangements of the monomers the larger aggregates, though mimicking the membrane amphiphilic environment, effectively resist against monomer straightening.

$N_{h}$ is actually the number of peptides that are in a fully functional fold. In that sense, the dependence in Figure 7a shows the development of the effective peptide concentration with time and can provide some theoretical background for the experimentally observed sigmoidal dependence of AMP’s activity on the concentration (see, e.g., [33], where the authors not only confirmed the importance of AMPs aggregation prior to their interaction with the membrane but also showed the necessity of some additional mechanism for explaining the aforementioned sigmoidal activity dependence).

It has been shown in multiple studies (e.g., [16]) that AMPs action depends on a threshold concentration, below which the peptides are unable to affect the target membrane. However, a number of authors suggested that the action of AMPs depends on their local surface, and not bulk concentration (see, e.g., [34,35]). Several different models for the mechanism of action of AMPs have been proposed, including the carpet model, the toroidal pores model and barrel stave pores model. They all depend on a threshold local surface concentration of the AMPs. Sengupta et al. [34] also demonstrated that aggregation at or near the membrane provides this critical local concentration and is necessary for the formation of transmembrane toroidal pores. However, their starting conformation included peptides, placed *“in the water phase close to one of the leaflets of an equilibrated DPPC bilayer”*. In our work, we demonstrate that this aggregation takes place very quickly, right after the AMPs are secreted and before reaching the membrane surface. The existence of localised isolated clusters and not isolated monomers in the bodily liquids prior to AMPs embedding in the bacterial membrane by no means contradicts the observed low concentration (in particular, of bombinin H2) in solution—it is only that the clusters need to be sparser than the isolated monomers.

Within aggregates, the peptides provide each other with an amphipathic environment mimicking the water–membrane interface that promotes further folding towards the biologically active shape—a single-helix structure, contrary to the case of isolated monomers. The latter might be viewed as representing a low peptide concentration situation. Identifying the critical concentration at which peptide assembly and functional folding promotion occur requires substantial computational resources and will be attempted in a separate study.

The above results support our hypothesis that it is the self-assembly process accompanied by aggregation-driven conformational changes into the biologically active fold that allow the AMPs to reach the target membrane in a fully functional state and to effectively exert their antimicrobial action.

## 4. Materials and Methods

### 4.1. Input Structures

**Figure 8:**
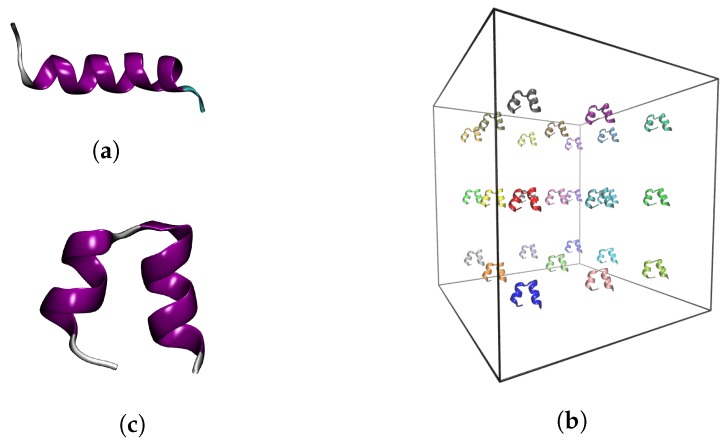
(**a**) Starting experimental structure of Bombinin H2. Input structures for the production simulation of: (**c**) monomeric bombinin H2; and (**b**) 27 bombinin H2 peptides in solution.

We used as a starting model the first frame in the NMR structure of bombinin in DPC micelles with PDB ID 2AP8 [29] (Figure 8a). There, the peptide is not in water solution, but in a DPC micelle environment where it adopts an all α-helical conformation. Once in the absence of a membrane, the peptide rapidly transitions to a helix-loop-helix state within the 10 ns of NPT equilibration simulation (Figure 8c).

This equilibrated structure was used in the isolated-monomer studies and also to build a solution of 27 bombinin H2 peptides in a cubic simulation box with an edge length of 15 nm. The distance between two peptides was 5 nm (Figure 8b).

### 4.2. MD Simulation Protocol

All simulations were performed with the MD simulation package GROMACS 2016.8 [36]. The CHARMM 27 force field was used for parameterisation of the peptides [37] in combination with the modified TIP3PS water model for the solvent [38]. The peptides were solvated in cubic boxes with a minimal distance to the box walls of 1.2 nm under periodic boundary conditions. Sodium and chlorine ions with a 0.15 mol/L concentration were added to all systems to neutralise their net charge and to ensure physiological salinity of the solution. The systems were energy minimised using the steepest descent with a maximum force tolerance of 10 kJ/(mol nm), followed by short 50 ps position-restraint simulations to equilibrate the solvent. Then, 10 ns isothermal-isobaric simulations were performed, in which the temperature was gradually increased to 310 K using v-rescale thermostat [39] with a coupling constant of 0.1 ps and pressure was equilibrated at 1 atm using a Parrinello–Rahman barostat [40,41] with a coupling constant of 2 ps.

The production MD simulations were also performed in the NPT-ensemble using the same thermo- and barostat parameters. The leapfrog integrator [42] was used with a time-step of 2 fs, whereas constraints were imposed on bonds between heavy atoms and hydrogens using the PLINCS algorithm [43]. Van der Waals interactions were smoothly switched off from a distance of 1.0 nm and truncated at 1.2 nm. Electrostatic interactions were treated using the smooth PME method [44] with a direct PME cut-off of 1.2 nm. Neighbour lists were constructed every 10 ps. Each production simulation had a duration of 2 μs. Trajectory frames were recorded every 100 ps.

### 4.3. Data Analysis

The trajectories were postprocessed and analysed using the standard GROMACS postprocessing and analysis tools, in particular those for RMSD, RMSF, SASA and R${}_{g}$ calculations and PCA and cluster analysis. The cluster and PC analyses were performed after all global translational and rotational movements were removed by least-square fitting to the starting conformation. Cluster analysis was performed using the gromos algorithm, with a cut-off of 0.3 nm. PCA was carried out in Cartesian coordinates on all 60 backbone atoms (N, Ca, and C). For secondary structure assignment the STRIDE algorithm [45] as implemented by the visualisation and manipulation package VMD [46] was used. All structural figures were also generated by VMD.

## 5. Conclusions

Understanding in detail the mechanism of action of AMPs is a crucial prerequisite for their optimisation and successful application in the clinical fight against multidrug-resistant bacteria. We consider that studying the behaviour of AMPs right after their secretion in the bodily fluids (water solution), prior to their interaction with the membranes of pathogenic cells, is a largely overlooked first step in this mechanism.

Here, we used molecular dynamics simulations to study the behaviour of a single and multiple bombinin H2 peptides in solution without the presence of a target membrane. We found that in monomeric form bombinin H2 preferably adopts a compact helix-loop-helix conformation and only occasionally visits the classical linear single-helix state. This simulation corresponds to a very low AMP concentration.

At higher concentrations, the bombinin H2 peptides self-associate into aggregates. In addition, the aggregation process drives a significant portion of the peptide chains to permanently transition from the compact helix-loop-helix to the linear single-helix conformational state, by providing the necessary amphypathic environment mimicking the membrane–solvent interface.

The simulations results confirm our initial hypothesis that bombinin H2 and probably other AMP in general do not exist in solution as isolated monomers that assemble into clusters upon interaction with a target membrane to form pores. They rather self-assemble in the solvent into aggregates that deliver a large portion of the peptides into a folded state to the cell membrane and so provide the critical local concentration of peptides in a fully functional form to exert their action of the lipid bilayer.

## Figures and Tables

**Table 1:** Transition of individual bombinin H2 chains from a helix-loop-helix to the single-helix state. The first column gives the chain ID; the second one shows the first moment, when this conformation was adopted; the third column enlists the IDs of the chains, which were within 5 Å of the linear helix at that moment; and the last column gives information on what type of an oligomer the transition took place in.

Chain ID	Time [ns]	Neighbours	Aggregate
A	903	E, F, M, W, Y	27-mer
B	917	G, H, K, N, P, S, Z	27-mer
D	931	H, O, P	27-mer
E	735	A, K, M, R, S, T	27-mer
F	404	A, C, E, I, M, R, U, W, Y	19-mer
J	1516	N, U, V, a	27-mer
V	25	a	Dimer
W	70	A, F, Y	Tetramer
a	22	V	Dimer

## Supplementary Materials

Supplementary materials can be found at https://www.mdpi.com/1422-0067/20/21/5450/s1.
