# Supplementary material for: Self-Association of Antimicrobial Peptides: A Molecular Dynamics Simulation Study on Bombinin
Source: Int J Mol Sci. 2019 Nov 1;20(21):5450. doi: 10.3390/ijms20215450 (PMC6862524; doi:10.3390/ijms20215450)
Supplement: Supplementary file 1 [file ijms-20-05450-s001.pdf]

*Article*

# Self-Association of Antimicrobial Peptides: A Molecular Dynamics Simulation Study on Bombinin

**Peicho Petkov [1], Elena Lilkova [2], Nevena Ilieva [2,3]* and Leandar Litov [1]**

1 Sofia University "St. Kliment Ohridski", Faculty of Physics, Atomic Physics Department, 5 J. Bouchier Blvd, 1164 Sofia, Bulgaria
2 Institute of Information and Communication Technologies at the Bulgarian Academy of Sciences, Acad. G. Bonchev Str., Block 25A, 1113 Sofia, Bulgaria
3 Institute of Informatics and Mathematics at the Bulgarian Academy of Sciences, Acad. G. Bonchev Str., Block 8, 1113 Sofia, Bulgaria
* Correspondence: nevena.ilieva@parallel.bas.bg

Version October 26, 2019 submitted to Int. J. Mol. Sci.

# Supplementary Material

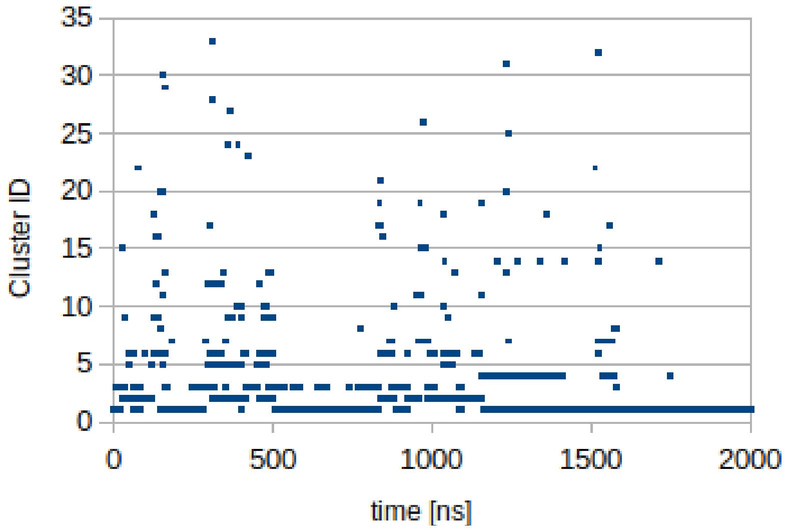


**Figure S 1.** Cluster population along the 2 $\mu$s trajectory of the single bombinin H2 monomer in water.

© 2019 by the authors. Submitted to *Int. J. Mol. Sci.* for possible open access publication under the terms and conditions of the Creative Commons Attribution (CC BY) license (http://creativecommons.org/licenses/by/4.0/).

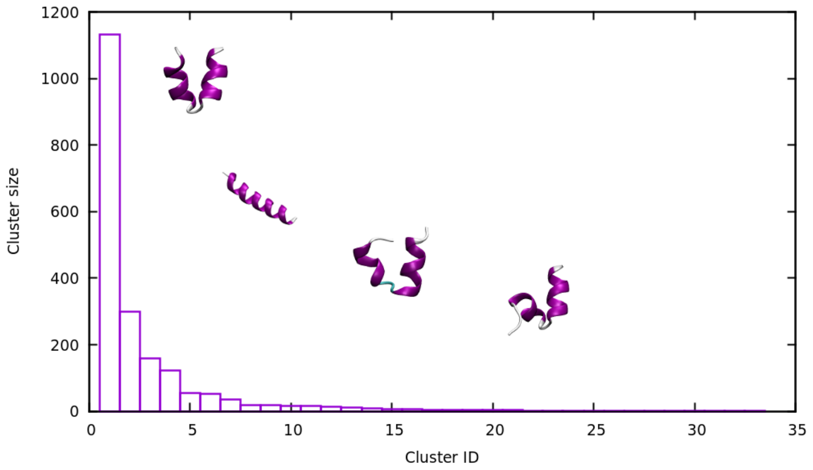


**Figure S 2.** Clusters' size and centroids of the four biggest clusters.

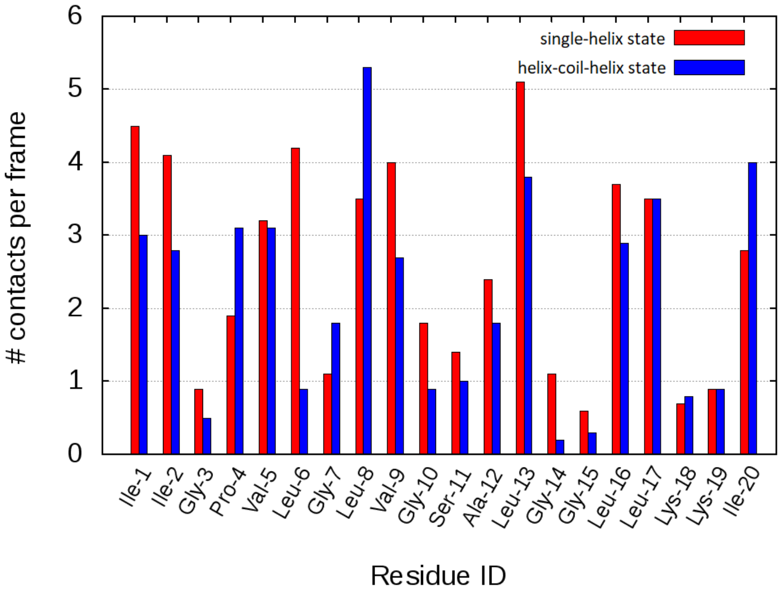


**Figure S 3.** Intermolecular contacts per frame for each of the residues in the bombinin H2 molecule in the single-helix and the helix-coil-helix states for the solution of 27 bombinin H2 monomers.

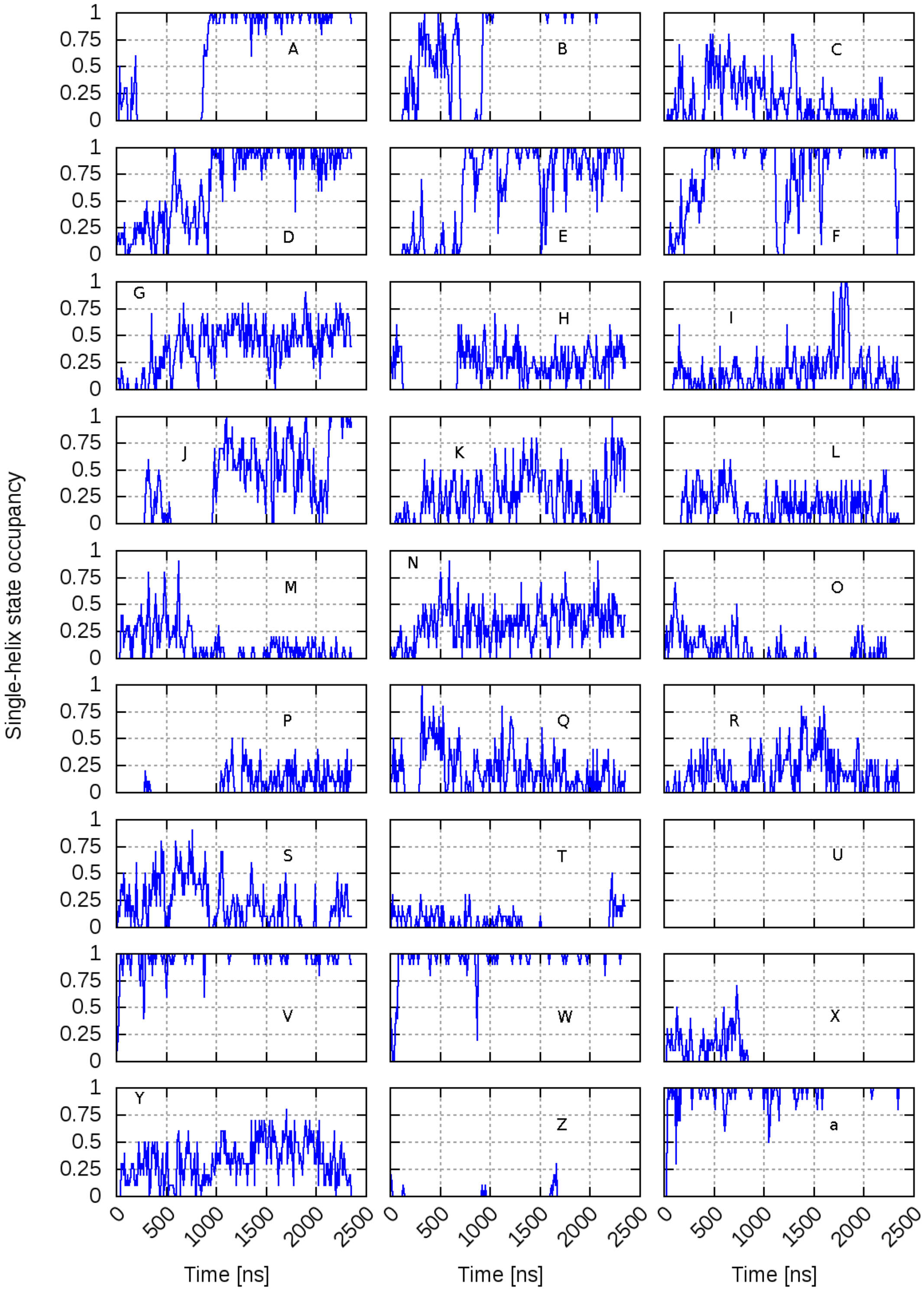


**Figure S 4.** Single-helix state occupancy for each of the 27 peptide chains.

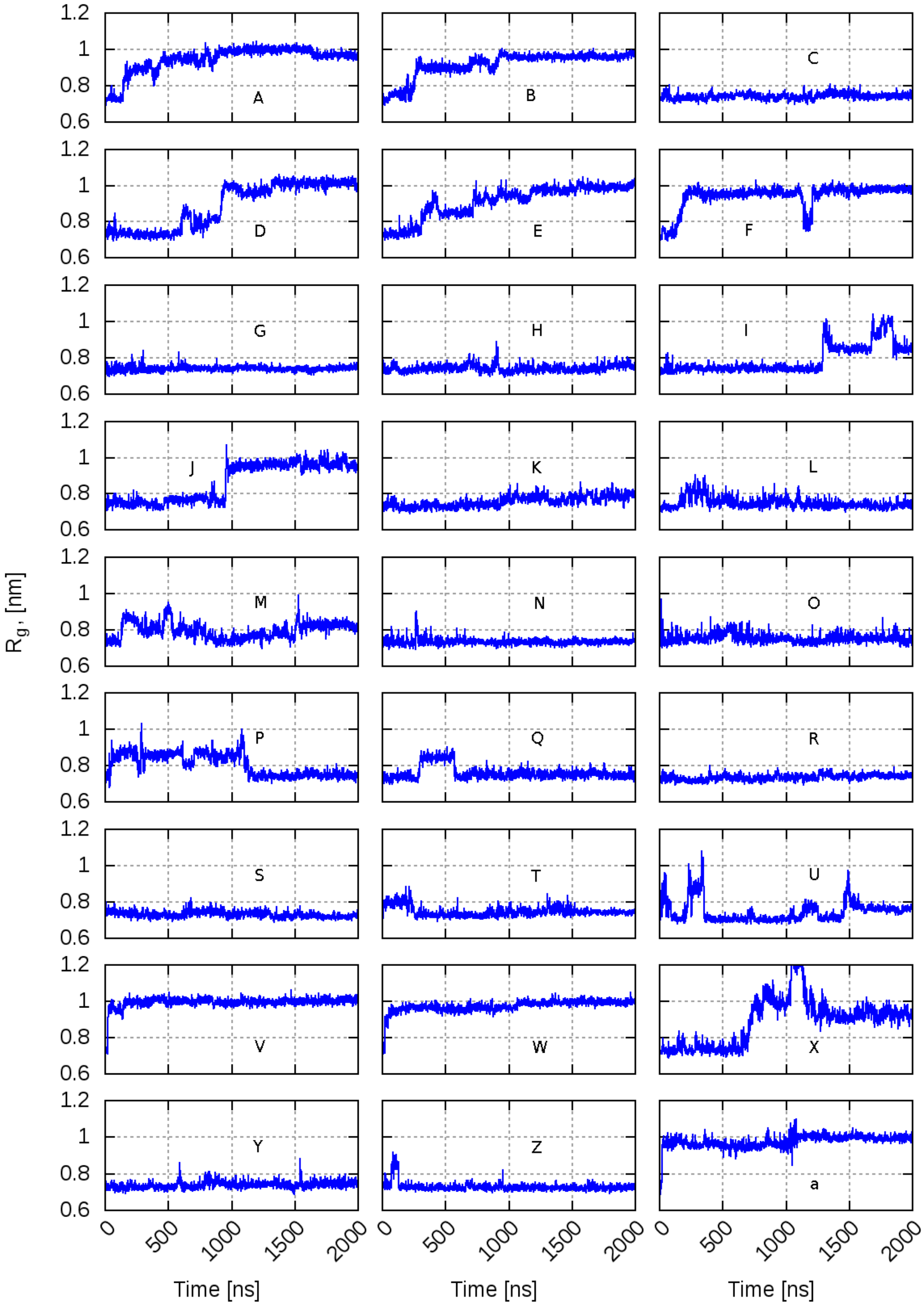


**Figure S 5.** Gyration radius for each of the 27 peptide chains.
